# The Royal Commission on Whiskey and Potable Spirits

**Published:** 1908-02-29

**Authors:** 


### The Hospital

A Journal of

The Medical Sciences and Hospital Administration.

New Series. No. 50, Vol. II. [No. 1122, Vol. XLIII.] SATURDAY, FEBRUARY 29, 1908.

### THE ROYAL COMMISSION ON WHISKEY AND POTABLE SPIRITS.

"Only a little more than a decade and a half has apsed since the Select Committee on British and oreign Spirits issued their Report. If we compare ıe minutes of reference to the Select Committee of 891 with those to the Royal Commission of 1908 'e find that for practicable purposes they are lentical. As we must be brief, we should describe the result of the 1891 Committee as negative. he Committee practically left the whiskey trade s it was; they found that the consumer early always got a wholesome drink, and that is own palate and his own purse protected him rom fraud. Sixteen years have passed since the bove investigation. During this time two changes f the first magnitude have occurred with regard to hiskey. Its consumption has been enormously ugmented, and the production of the mild or lended whiskey in proportion to the highly avoured pot-still or self-whiskey has very much ncreased. In fact the production of so-called silent pirit or whiskey produced from malt and other grain n a patent still has developed from a relatively small nto a very great industry.

The increase in the consumption of whiskey is argely due to the medical profession. During the last twenty years doctors have recommended whiskey both as an ordinary dietetic beverage, chiefly on account of its low acidity as compared with wine and beer, and also as a medicine, chiefly because of the difficulty experienced a few years ago of getting good brandy and the price of the latter. The main ingredient to which the dietetic and medicinal properties of whiskey are due, and the chief constituent of it of interest to the medical profession, is alcohol, and it must at once be emphasised that whiskey is one of the most convenient vehicles for the administration of alcohol in a pure form, and almost in any degree of dilution that may be required. Whiskey is, however, not pure alcohol. It contains in addition more or less of certain by-products which impart to it certainly flavour and character, and possibly, perhaps even probably, dietetic and medicinal properties. Speaking generally, Highland Scotch pot-still whiskey and Irish pot-still whiskey contain more of these by-products than Lowland malt whiskey, and this latter more of them than blended whiskey—that is, whiskey consisting of different proportions of pot-still and patent-still whiskey, and this latter more than patent-still whiskey.

Some two years ago The Hospital appointed an Analytical Commission upon whiskey, and in the report of this Commission will be found a description of the method of manufacture and of the chemical and therapeutic properties of whiskey. It may briefly be stated here that the manufacture of whiskey consists essentially of four specific processes, namely malting, mashing, fermenting, and distilling. Differences in each of these processes produce a difference in the finished article. Those, however, which technically come most prominently into consideration are a difference in the constituents from which the infusion subsequently to be fermented, or the wort, is made, and a difference in the process by which the fermented wort is distilled. In Scotch pot-still distilleries the mash always consists of malted barley alone. In Irish pot-still distilleries the malted barley is frequently mixed with other grain. In patent-still distilleries, the mash practically always consists of malted barley mixed with other grain. Speaking generally, the more malted barley the mash contains the greater is the cost of production, and hence to obtain a reasonable profit the higher must be the price of the finished article. As we said above, even with the same constituents used for the mash, the finished product as it leaves the still contains more by-products in the case of the pot than in the case of the patent still. These by-products, from the point of view of the consumer, may roughly be divided into two classes, those that give a pleasant taste and character to the whiskey, and possibly possess advantageous therapeutical properties, and those which are disagreeable and nauseating to the palate, and possibly give rise to injurious physiological effects. By maturation or ageing these latter products disappear, and the former ones become reinforced. The keeping or maturation of whiskey is expensive to the distiller, because he has to pay the rent of bonding and further loses interest upon his capital. Patent-still whiskey and blended whiskey, speaking generally, require less ageing to become pleasant to the palate than pot-still whiskey. There is also evidence to show that ill-matured potstill whiskey may produce bad physiological effects, and also that whiskey or spirit distilled in a patent-still, but made from a mash containing diseased grain or other fermentable substances, may likewise be injurious to health, in that some of the by-products of fermentation may be of an alkaloidal nature and volatile, thus passing over into the finished spirit. There is very little actual evidence upon either of these subjects, but this part of the question will certainly engage the attention of the Committee.

From these considerations four facts will be clear. First, that the term "whiskey" as at present used comprises a number of beverages produced by different methods from different materials, and at different cost of production, but all resembling each other sufficiently to justify the practical public in designating each of them as whiskey. Second, that a consumer who asks for whiskey and likes a mild, characterless whiskey pays the same price for an article, that really costs much less, as does the consumer who likes a full-bodied, heavy whiskey. Third, that it is possible that the public may be prejudiced in its health by drinking an immature whiskey. Lastly, that the doctor who prescribes simply whiskey for his patient is prescribing by no means a therapeutic entity, as his patient may get pot-still whiskey, blended whisky, or patent-still whiskey. These considerations are essentially those which will engage the attention of the Commission, and it is to be hoped that their labours will result in raising the standard of whiskey obtainable at a reasonable price, both as a dietetic beverage and as a medicinal preparation.

We cannot here enter into the question of brandy, rum, and gin, all of which are obviously comprised in the minutes of reference, and are almost of equal importance. The Commission is essentially a Commission of experts, the trade as such being practically unrepresented upon it, and in this respect it differs very much from its predecessors.

